# Effective natural inhibitors targeting IGF-1R by computational study

**DOI:** 10.18632/aging.204117

**Published:** 2022-06-09

**Authors:** Xinyu Wang, Pengcheng Zhou, Liangxin Lin, Bo Wu, Zhaoyu Fu, Xing Huang, Dong Zhu

**Affiliations:** 1 Department of Orthopaedics, The First Bethune Hospital of Jilin University, Changchun, China; 2 Department of Radiology, Jilin Province People’s Hospital, Changchun, China

**Keywords:** Ewing sarcoma, IGF-1R, drug therapy, natural compounds, virtual screening

## Abstract

IGF-1R belongs to a tyrosine kinase family and is currently a newly discovered drug target. IGF-1R inhibitors can bind directly to IGF-1R to achieve the effect of inhibiting the function of IGF-1R. At present, IGF-1R inhibitors have good clinical effects on Ewing sarcoma in the clinic. In this article, we screened compounds capable of inhibiting IGF-1R function through computer-aided virtual technology. First, some molecules with good docking properties for IGF-1R can be screened by LibDock. Then, ADME analysis (adsorption, distribution, metabolism, and excretion) and toxicity indicators were performed. The mechanism of binding and the binding affinity in the middle of IGF-1R and ligand were verified using molecular docking. Ultimately, the stability of ligand-receptor complex was evaluated using molecular dynamics simulations.

In line with the results, two natural compounds ZINC000014946303 and ZINC000006003042 were found in the ZINC database, potential effective inhibitors of IGF-1R. ZINC000014946303 and ZINC000006003042 can bind to IGF-1R with high binding affinity as predicted by molecular docking. It was also found that they are not hepatotoxic, with less developmental toxicity potential, rodent carcinogenicity, Ames mutagenicity, and high tolerance to cytochrome P4502D6. Hereby, this study aimed to screen out ideal compounds that have inhibitory effects on IGF-1R from the drug library and, at the same time, provide a direction for the future development of IGF-1R inhibitors.

## INTRODUCTION

Ewing sarcoma is a type of primary malignant bone tumor with the second incidence among children and adolescents, and its proportion in primary bone tumors is 6% to 8% [1]. The disease is characterized by a short disease course, rapid metastasis, and high malignancy. The current primary treatments for Ewing sarcoma are surgery alone, radiotherapy, and single-agent chemotherapy [2]. However, none of the treatments have favorable outcomes, and most patients will die within 2 years, with 5-year survival rates of no more than 10% [3]. Therefore, there is a need to develop more effective treatment strategies for Ewing sarcoma.

Ewing sarcoma is characterized by translocation of chromosomes 22 and 11, resulting in fusion of the corresponding EWS gene and FLI1 gene, thus expressing the fusion gene EWS-FLI1 [4]. The fusion gene was then translated to produce the protein EWS-FLI1, which directly induced or inhibited the enhancer recombinant Ewing sarcoma gene regulatory circuit. This is why Ewing sarcoma is so common [5]. IGF-1R (Insulin-like growth factor 1 receptor) belongs to a tyrosine kinase family. It is often activated and overexpressed in Ewing sarcoma. IGF-1R exerts an enormous function on the genesis and evolution of Ewing sarcoma [6]. Incidence rates of Ewing sarcoma and endogenous IGF-1 levels peaked around puberty [4]. IGF-1R signaling has an effect on endogenous IGF-1, and the fusion gene EWS-FLI1 is present in Ewing sarcoma. This suggests that the IGF-1R signal may have a synergistic effect on fusion gene EWS-FLI1, thus driving the occurrence of Ewing sarcoma.

In Ewing sarcoma, IGF-1R binds to IGF to initiate SRC, MAPK, FAK, and other signaling pathways to synergistically stimulate the proliferation, survival, and migration of cancer cells in Ewing sarcoma [7]. Studies have shown that the inhibitors of tyrosine kinase and anti-IGF-1R antibodies have good therapeutic potential in Ewing sarcoma [8]. Clinical trials to evaluate the efficacy of anti-IGF-1R monoclonal antibodies are also underway [9]. The clinical activity of anti-IGF-1R mAb has been demonstrated to have a sustained response in a few patients with specific tumor types such as Ewing sarcoma and thymoma. Nonetheless, in other cancer patients, anti-IGF-1R mAb have no significant therapeutic effect [9]. TAE226 is a tyrosine kinase inhibitor that acts on IGF-1R and FAK. TAE226, in combination with conventional chemotherapy, improved the prognosis of patients with recurrent and metastatic Ewing sarcoma and substantially inhibited local growth and metastasis of primary tumors in Ewing sarcoma. Disappointingly, TAE226 demonstrated high cytotoxicity in Ewing sarcoma cells [10]. Hereby, finding the optimal IGF-1R inhibitors can provide new ideas and options for treating Ewing sarcoma.

This study aims to screen out novel and promising IGF-1R targeting agents which can treat Ewing sarcoma. Natural products can play the function of lead compounds. Subsequently, the products are modified and converted to become a new drugs. It is also becoming a new drug source for the pharmaceutical industry [8, 11]. This study will use various chemical methods and structural biology (such as molecular docking, virtual screening, etc.) in the screening and identification of compounds that can inhibit the function of IGF-1R. In the meantime, we will further study the metabolism, absorption level, distribution characteristics, and side effects of these compounds. The candidate compounds and their pharmacological properties were listed, which provided a direction for exploiting IGF-1R inhibitors.

## RESULTS

### Virtual filtering of ZINC15 database for IGF-1R

ZINC is a free public resource for ligand discovery. The database contains over twenty million commercially available molecules in biologically relevant representations that may be downloaded in popular ready-to-dock formats and subsets. The Web site also enables searches by availability, bioactive and drugs, biogenic, reactivity, and others. ZINC connects purchasable compounds to high-value ones such as metabolites, drugs, natural products, and annotated compounds from the literature [12, 13]. Natural products have evolved over millions of years and acquired a unique chemical diversity, resulting in the diversity of their biological activities and drug-like properties. Therefore, even before the rise of modern chemical pharmacology, natural products have been used for centuries as components of traditional medicines, particularly as active components of herbal remedies. In modern pharmacology too, natural products have become one of the most critical resources for developing new lead compounds and scaffolds [14]. Therefore, we chose to select ligands from the subset of natural products in the biogenic classification of the ZINC database, and these ligands must have been named and for sale, which can facilitate our experimental studies. In the end, we selected 17931 ligands for follow-up research through the screening of the above conditions.

The structure of IGF-1R will be used to construct the receptor, and then we will perform a LibDock docking operation between the receptor and 17931 ligands. By molecularly docking 17931 ligands with IGF-1R one by one, 5169 ligands that could be successfully docked with it were selected. ZINC38433168 is a new class of IGF-1R inhibitors related to hydantoins that was discovered through high-throughput screening [15]. It can combine with IGF-1R, making it difficult for IGF-1R to activate various signal pathways so as to achieve the purpose of treating Ewing sarcoma [15]. Libdock is a fast molecular docking tool for fast and accurate virtual screening of large-scale databases. The higher the score, the better the effect of molecular docking. In accordance with LibDock score, the compounds with the top 20 scores proceeded to the next step (Table 1).

**Table 1:** Top 20 ranked compounds with higher LibDock scores.

**Number**	**Compounds**	**LibDock score**
1	ZINC000014767731	121.452
2	ZINC000032840969	124.667
3	ZINC000043016594	125.121
4	ZINC000028968107	121.189
5	ZINC000003831332	130.435
6	ZINC000014946303	141.977
7	ZINC000169363256	127.727
8	ZINC000006003042	132.163
9	ZINC000040865806	126.521
10	ZINC000049088142	123.691
11	ZINC000044086691	131.832
12	ZINC000013451932	124.17
13	ZINC000002526389	129.412
14	ZINC000002528510	126.077
15	ZINC000027646038	133.826
16	ZINC000027646060	121.422
17	ZINC000049878068	121.451
18	ZINC000011616636	143.901
19	ZINC000001916008	132.557
20	ZINC000100772345	124.35

### ADME properties of ligands

The ADME module predicted the pharmacological properties of 17931 ligands and the reference drug ZINC38433168. Pharmacological properties listed in Table 2 include water-soluble level, plasma protein binding (PPB) properties, liver toxicity, blood-brain barrier (BBB) level, binding to CYP2D6, and intestinal absorption level. By definition of water-soluble, it is found that most compounds can be soluble in water. For the blood-brain barrier, ZINC000040865806 and ZINC000013451932 have low permeability, ZINC000027646038 and ZINC000027646060 have high permeability, and the levels of all other compounds are uncertain. 17 compounds and the reference drug ZINC38433168 are predicted to act as non-CYP2D6 inhibitors that significantly affect drug metabolism. 8 compounds were also found to have no hepatotoxicity, similar to the reference drug ZINC38433168. 6 compounds were found to have good intestinal absorption characteristics. 9 compounds were strongly absorbent based on the plasma protein binding properties.

**Table 2:** Adsorption, distribution, metabolism, and excretion properties of compounds.

**Number**	**Compounds**	**Solubility level**	**BBB level**	**CYP2D6**	**Hepatotoxicity**	**Absorption level**	**PPB level**
1	ZINC000014767731	0	4	1	1	3	0
2	ZINC000032840969	2	4	1	1	3	1
3	ZINC000043016594	1	4	1	0	2	0
4	ZINC000028968107	1	4	0	0	3	0
5	ZINC000003831332	0	4	1	1	3	0
6	ZINC000014946303	0	4	1	0	3	1
7	ZINC000169363256	3	4	1	1	1	1
8	ZINC000006003042	2	4	1	0	1	0
9	ZINC000040865806	3	3	1	1	0	1
10	ZINC000049088142	1	4	1	0	2	1
11	ZINC000044086691	1	4	1	1	3	0
12	ZINC000013451932	3	3	1	0	0	0
13	ZINC000002526389	2	4	0	0	0	0
14	ZINC000002528510	2	4	0	0	0	0
15	ZINC000027646038	3	1	1	1	0	1
16	ZINC000027646060	3	1	1	1	0	1
17	ZINC000049878068	1	4	1	1	3	1
18	ZINC000011616636	2	4	1	1	3	1
19	ZINC000001916008	1	4	1	1	3	0
20	ZINC000100772345	3	4	1	1	1	0
21	ZINC38433168	-	-	1	0	-	0

BBB, blood-brain barrier; CYP2D6, cytochrome P-450 2D6; PPB, plasma protein binding.

Aqueous-solubility level: 0, extremely low; 1, very low, but possible; 2, low; 3, good.

BBB level: 0, very high penetrant; 1, high; 2, medium; 3, low; 4, undefined.

CYP2D6 level: 0, noninhibitor; 1, inhibitor.

Hepatotoxicity: 0, nontoxic; 1, toxic.

Human-intestinal absorption level: 0, good; 1, moderate; 2, poor; 3, very poor.

PPB: 0, absorbent weak; 1, absorbent strong.

At the same time, we should also attach great importance to the safety of natural compounds. As far as the safety of the compounds is concerned, we used the TOPKAT module to study the toxicity of these 20 compounds and the reference drug ZINC38433168, such as rodent carcinogenicity, Ames mutagenicity, and potential characteristics of developmental toxicity (Table 3). Among the results, it was found that 7 compounds were nonmutagenic, and 5 compounds had no developmental toxicity. The results in Table 3 predict that the reference drug ZINC38433168 has high rodent carcinogenicity in mice but no rodent carcinogenicity in rats. Considering all of the above results, ZINC000014946303 and ZINC000006003042 have no hepatotoxicity.

**Table 3:** Toxicities of compounds.

**Number**	**Compounds**	**Mouse NTP**		**Rat NTP**		**Ames**	**DTP**
		**Female**	**Male**	**Female**	**Male**		
1	ZINC000014767731	0	1	1	0	1	1
2	ZINC000032840969	0	0.961	0	0.692	0.359	0
3	ZINC000043016594	0	1	0.001	0.009	0	1
4	ZINC000028968107	1	0.021	0.06	0.997	1	1
5	ZINC000003831332	1	1	0	0.993	0	1
6	ZINC000014946303	0.991	1	0	1	0	0.007
7	ZINC000169363256	1	1	1	0.176	1	0.01
8	ZINC000006003042	0	1	0	0.001	1	1
9	ZINC000040865806	0	0.065	0.989	0.11	0	0.791
10	ZINC000049088142	0	1	1	0	0	0
11	ZINC000044086691	1	0	0.987	0.998	0.004	1
12	ZINC000013451932	1	0	0	0.685	0	0.999
13	ZINC000002526389	0.999	0.036	0	0.999	0.999	0.769
14	ZINC000002528510	0.999	0.036	0	0.999	0.999	0.769
15	ZINC000027646038	0	0	0	0	0.506	0
16	ZINC000027646060	0	0	0	0	0.482	0
17	ZINC000049878068	0.837	0.938	0	1	0	0.906
18	ZINC000011616636	0	1	1	1	1	1
19	ZINC000001916008	0	1	1	0	1	1
20	ZINC000100772345	0	0.999	0	1	1	0
21	ZINC38433168	0.982	1	0	0	0.052	0.993

NTP, U. S. National Toxicology Program; DTP, developmental toxicity potential.

NTP<0.3(noncarcinogen);>0.8(carcinogen).

Ames<0.3(nonmutagen);>0.8(mutagen).

DTP<0.3(nontoxic);>0.8(toxic).

**Figure 1:**
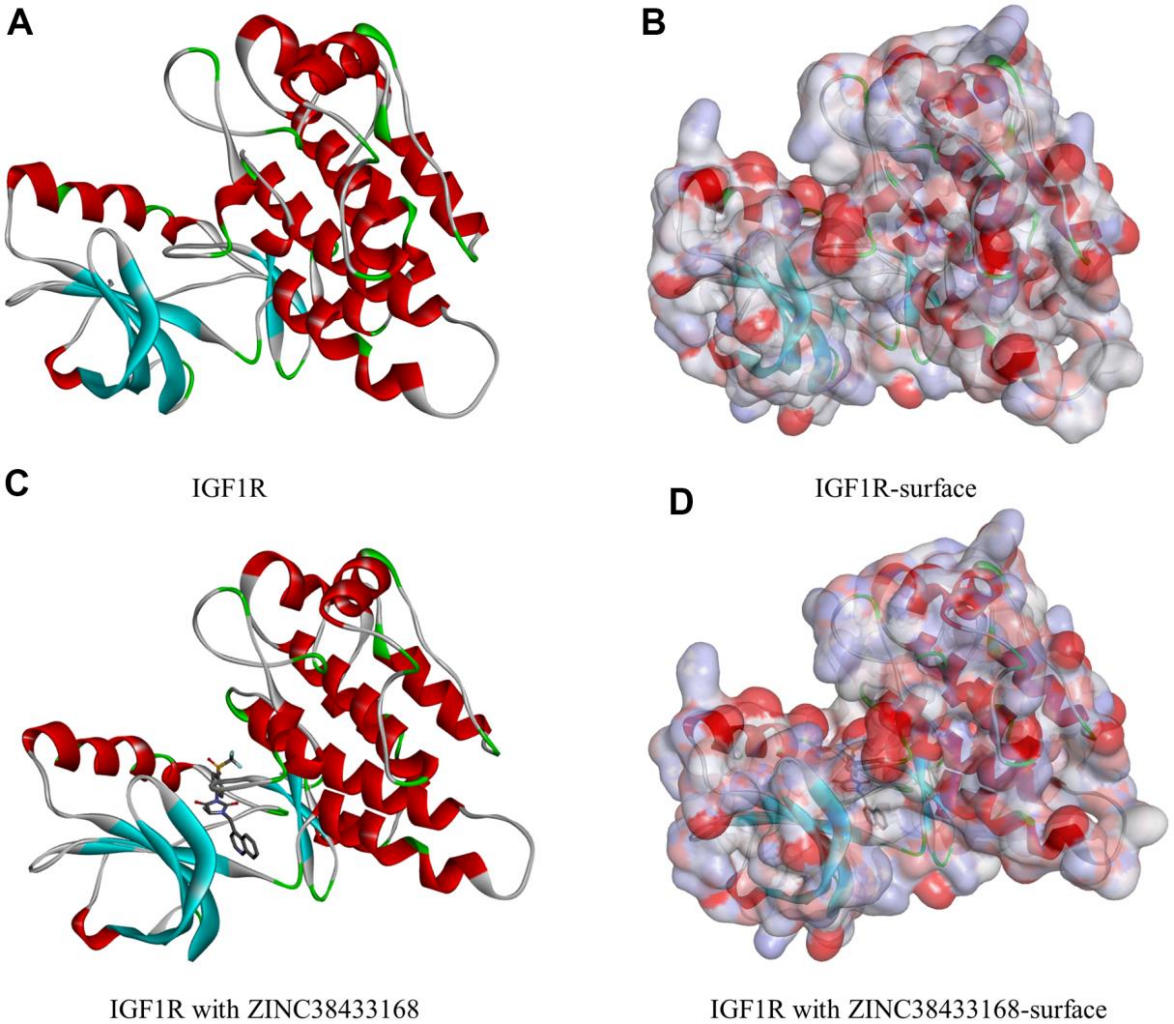
**Molecular structure of IGF-1R.** (**A**) Initial molecular structure. (**B**) Surface of binding area added. Blue represents positive charge, and red represents negative charge. (**C**) Molecular structure after IGF-1R and ZINC38433168 binding. (**D**) Surface of IGF-1R and ZINC38433168 binding area added. Blue represents positive charge, and red represents negative charge.

**Figure 2:**
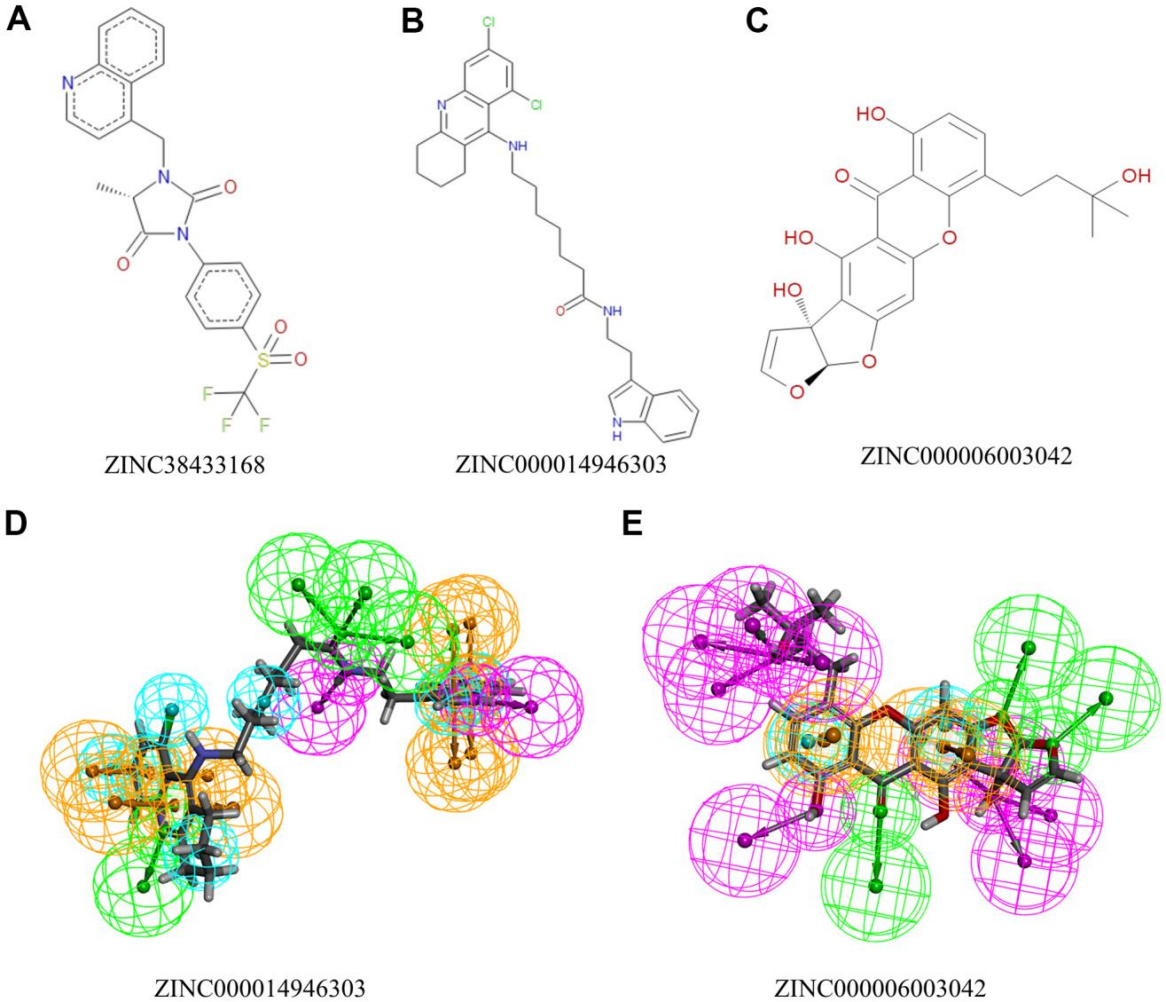
**The 2D structures of natural compounds selected from virtual screening by ChemDraw.** And Pharmacophore predictions using 3D-QSAR. (**A**) ZINC38433168; (**B**) ZINC000014946303; (**C**) ZINC000006003042. (**D**) ZINC000014946303: Green represents hydrogen acceptor, blue represents hydrophobic center, purple represents hydrogen donor, and yellow represents ring aromatic. (**E**) ZINC000006003042: Green represents hydrogen acceptor, blue represents hydrophobic center, purple represents hydrogen donor, and yellow represents ring aromatic.

Moreover, their rodent carcinogenicity, Ames mutagenicity, and developmental toxicity are at a low level compared to the remaining compounds. Overall, we can identify ZINC000014946303 and ZINC000006003042 as safe drugs. ZINC000014946303 and ZINC000006003042 and the reference drug ZINC38433168 have similarities in their chemical structures and were selected for the following study (Figures 1, 2).

### Ligand-binding analysis

**Figure 3:**
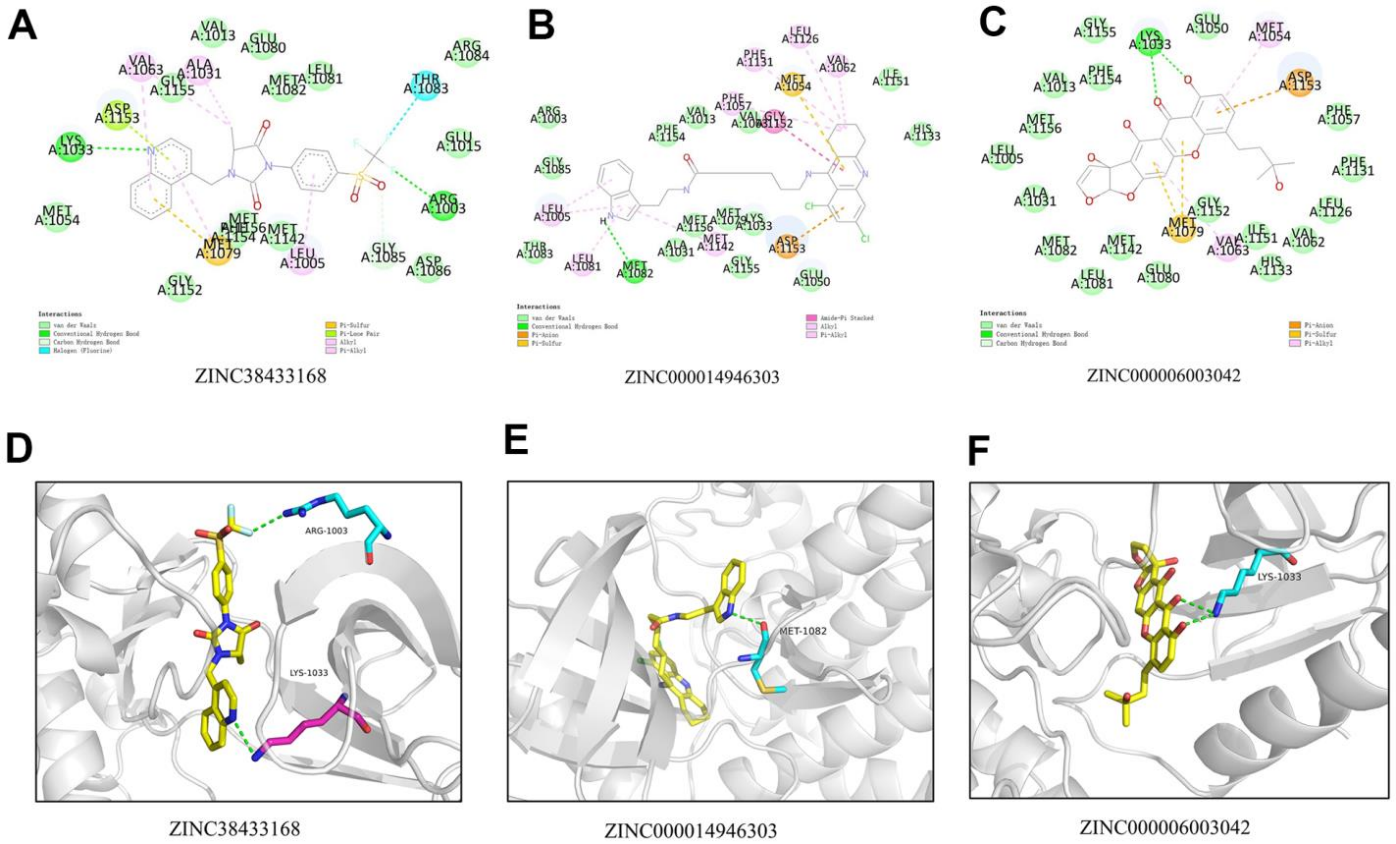
(**A**) 2D intermolecular interaction diagram of the ZINC38433168/IGF-1R complex. (**B**) 2D intermolecular interaction diagram of the ZINC000014946303/IGF-1R complex. (**C**) 2D intermolecular interaction diagram of the ZINC000006003042/IGF-1R complex. (**D**) The residue and the hydrogen bond in the ZINC38433168. (**E**) The residue and the hydrogen bond in the ZINC000014946303. (**F**) The residue and the hydrogen bond in the ZINC000006003042.

**Figure 4:**
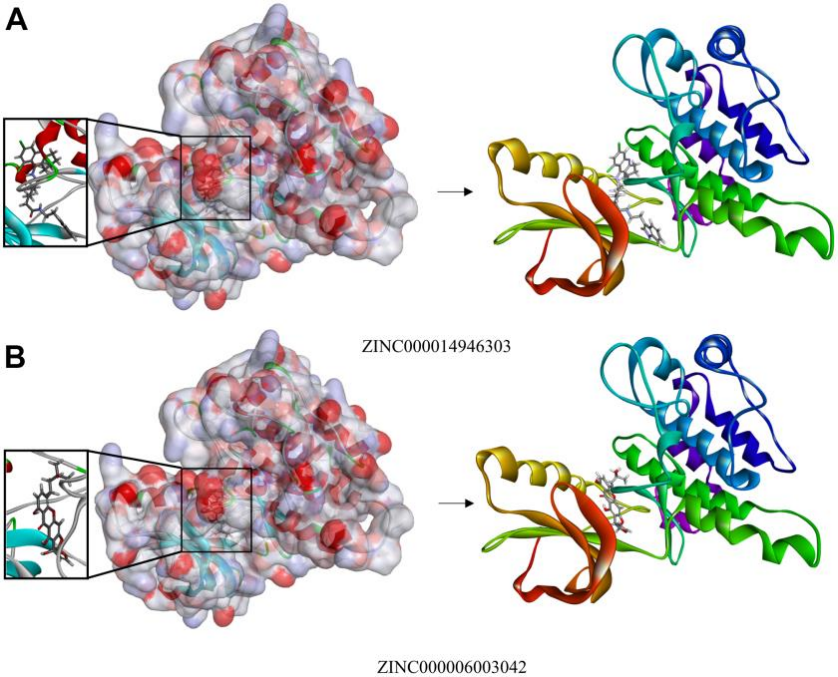
(**A**) Schematic drawing of the interactions between IGF-1R and ZINC000014946303. (**B**) Schematic drawing of the interactions between IGF-1R and ZINC000006003042.

We will use IGF-1R to study the ligand binding mechanism of candidate compounds. Firstly, we docked ZINC000014946303, ZINC000006003042, and ZINC38433168 into the molecular structure of IGF-1R through the CDOCKER module, calculated and displayed the CDOCKER potential energy. The reference drug ZINC38433168 has higher CDOCKER potential energy than ZINC000014946303 and ZINC000006003042 as calculated by the CDOCKER module (Table 4). In contrast to the reference drug ZINC38433168, IGF-1R has a higher binding affinity for ZINC000014946303 and ZINC000006003042. The hydrogen bond and π-related interactions were studied by structural calculation (Figures 2, 3). Characteristic pharmacodynamic masses refer to the active conformation that produces geometric and energy matching when the drug molecule interacts with the receptor target. As shown in Figures 2, 3, there were 21 Characteristic pharmacodynamic masses in ZINC000014946303 and 32 Characteristic pharmacodynamic masses in ZINC000006003042. In the complex, they form several pairs of π-related interactions and hydrogen bonds with IGF-1R. In Tables 5, 6, 3 hydrogen bond pairs were found in the complex formed by the combination of ZINC000006003042 and IGF-1R, by O11 of ZINC000006003042 with LYS1033:HZ1 of IGF-1R, by O23 of ZINC000006003042 with LYS1033:HZ3 of IGF-1R, by O23 of ZINC000006003042 with LYS1033:HE2 of IGF-1R (Table 5). We also show the residue and the hydrogen bond in the structural figures (Figure 3D–3F). ZINC000006003042 and IGF-1R also formed a 4-pair π-related interaction, by 2 pairs of MET1079 of IGF-1R with ZINC000006003042, by 1 pair of MET1054 of IGF-1R with compound, by 1 pair of VAL1063 of IGF-1R with compound (Table 6). ZINC000014946303 and IGF-1R formed 9 pairs of π-related interaction and 1 pair of hydrogen bonds. When the reference drug ZINC38433168 is combined with IGF-1R, 6 pairs of π-related interactions and 3 pairs of hydrogen bonds will be formed. The interaction between ZINC000014946303 and ZINC000006003042 and IGF-1R is shown in Figure 4.

**Table 4:** CDOCKER potential energy of compounds with IGF1R.

**Compounds**	**-CDOCKER potential energy (kcal/mol)**
ZINC000014946303	63.4812
ZINC000006003042	52.3393
ZINC38433168	49.0370

**Table 5:** Hydrogen bond interaction parameters for each compound with IGF1R residues.

**Receptor**	**Compound**	**Donor atom**	**Receptor atom**	**Distances (Å)**
3o23	ZINC000014946303	ZINC000014946303:H67	MET1082:O	2.22953
			
	ZINC000006003042	LYS1033:HZ1	ZINC000006003042:O11	1.81577
		LYS1033:HZ3	ZINC000006003042:O23	2.42906
			
	ZINC38433168	ARG1003:HH22	3O23:F28	2.29
		LYS1033:HZ3	3O23:N29	2.58

**Table 6:** π-related interaction parameters for each compound with IGF1R.

**Receptor**	**Compound**	**Donor atom**	**Receptor atom**	**Distances (Å)**
3o23	ZINC000014946303	GLY1152:C,O;ASP1153:N	ZINC000014946303	4.52
		VAL1062	ZINC000014946303	4.84
		LEU1126	ZINC000014946303	5.01
		PHE1057	ZINC000014946303	5.48
		PHE1131	ZINC000014946303	5.43
		ZINC000014946303	LEU1005	4.62
		ZINC000014946303	LEU1081	5.17
		ZINC000014946303	MET1142	5.23
		ZINC000014946303	LEU1005	4.33
	ZINC000006003042	ZINC000006003042	MET1054	5.34
		ZINC000006003042	MET1079	5.48
		ZINC000006003042	VAL1063	4.92
		ZINC000006003042	MET1079	4.75
	ZINC38433168	ALA1031	3O23:C14	3.77
		3O23:C14	VAL1063	4.57
		3O23	LEU1005	4.34
		3O23	MET1079	5.32
		3O23	VAL1063	5.36
		3O23	MET1079	5.06

### Molecular dynamics simulations

**Figure 5:**
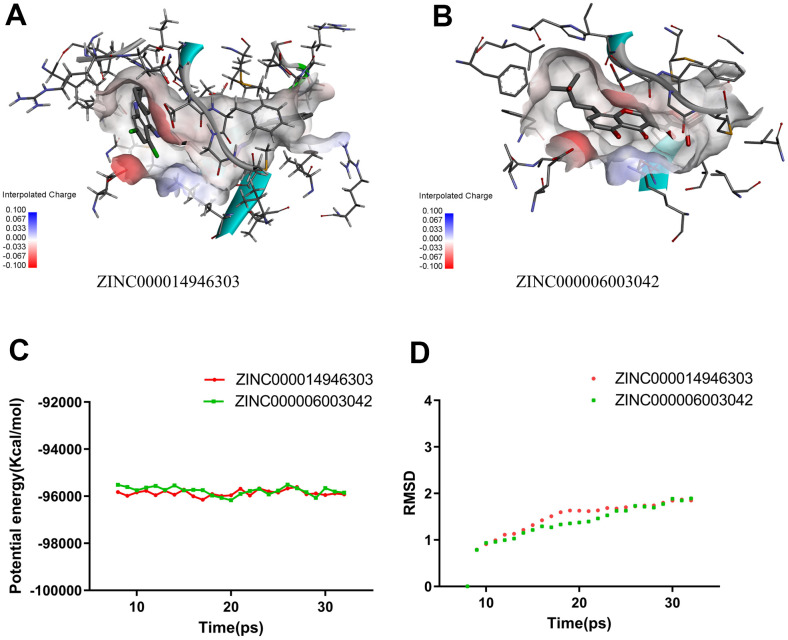
**Schematic drawing of interactions between ligands and IGF-1R.** (**A**) ZINC000014946303-IGF-1R complex. (**B**) ZINC000006003042-IGF-1R complex. (**C**) Potential Energy. (**D**) Average backbone RMSD.

We use molecular dynamics simulation to evaluate the stability of the ligand-IGF-1R in the natural environment and use the CDOCKER module to carry out molecular docking experiments and then obtain the original conformation. The potential energy and RMSD images for each complex are listed in Figure 5. After 20 ps, the tracks of ZINC000014946303 and ZINC000006003042 come into equilibrium. In time, the potential energy and RMSD of these 2 complexes tend to be stable. In brief, both ZINC000014946303 and ZINC000006003042 can bind to IGF-1R to form complexes that can exist stable in the natural environment inhibiting IGF-1R.

### ZINC000014946303 and ZINC000006003042 reduces the proliferation of Ewing sarcoma cells

**Figure 6:**
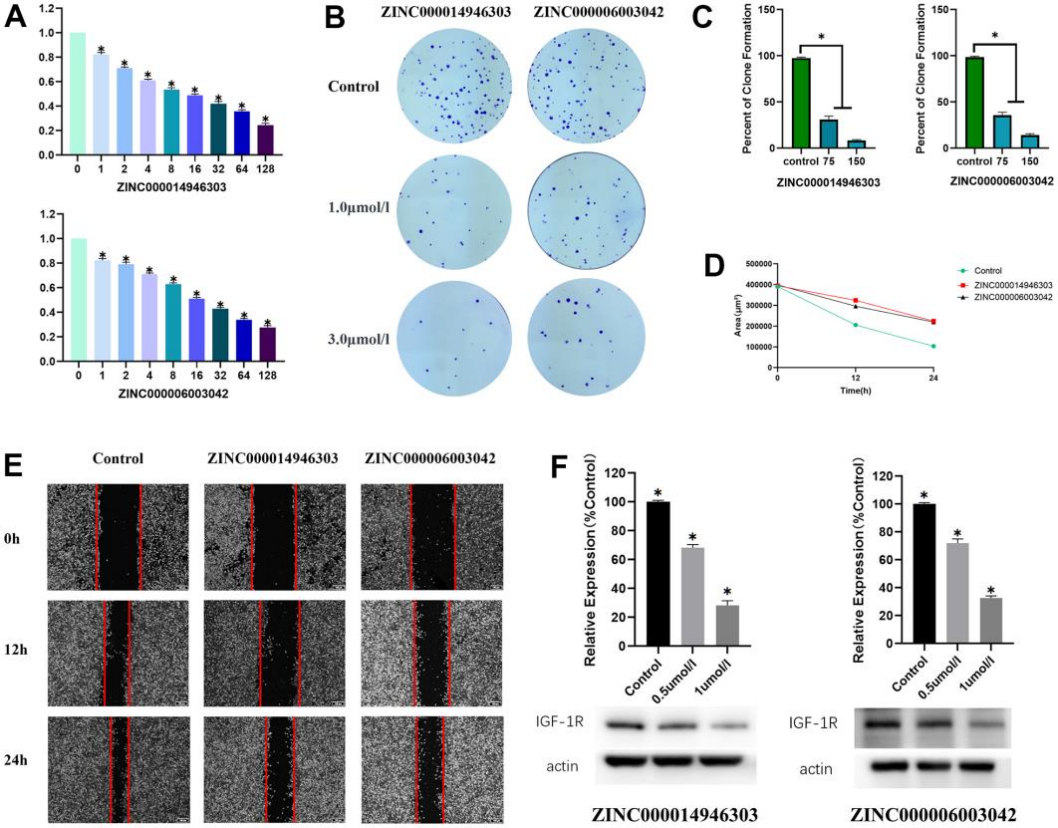
**Anti- Ewing sarcoma effects of ZINC000014946303 and ZINC000006003042.** (**A**) Cellular viability of Ewing sarcoma cells treated with ZINC000014946303 and ZINC000006003042. (**B**) Colony formation assay results demonstrate the anti-proliferative effects of ZINC000014946303 and ZINC000006003042 in Ewing sarcoma cells. (**C**) Numbers of clones formed by Ewing sarcoma cells. (**D**) Scratch assay results demonstrate that ZINC000014946303 and ZINC000006003042 suppressed the migration of Ewing sarcoma cells. (**E**) Images from the wound-healing assay representing the migration capacity of Ewing sarcoma cells. Anti- Ewing sarcoma effects of ZINC000014946303 and ZINC000006003042 targeting IGF-1R. (**F**) Results of western blot; Relative expression of IGF-1R(%Control).

We examined the effect of ZINC000014946303 and ZINC000006003042 on Ewing sarcoma cells *in vitro*. A 3-(4,5-dimethylthiazol-2-yl)-2,5-diphenyltetrazolium bromide (MTT) assay showed that the viability of RD-ES cells decreases as the concentration of the drug increases (P < 0.05) (Figure 6A). In a colony formation assay, RD-ES cells treated with ZINC000014946303 and ZINC000006003042 exhibited lower clonogenicity in both number and size than the control group cells (Figure 6B, 6C).

### ZINC000014946303 and ZINC000006003042 reduces the migration of Ewing sarcoma cells

The effect of ZINC000014946303 and ZINC000006003042 on Ewing sarcoma cells invasion and migration is determined by a scratch assay. After 24 h, the scratch area decreased significantly in the control group, but only slightly in the ZINC000014946303 group and the ZINC000006003042 group (Figure 6D, 6E). Although the scratch area in the control group, the ZINC000014946303 group, and the ZINC000006003042 group all decreased over time, the reduction was greater in the control group. These results suggest that ZINC000014946303 and ZINC000006003042 reduce the migration of Ewing sarcoma cells.

### ZINC000014946303 and ZINC000006003042 reduce IGF-1R expression in Ewing sarcoma cells

To verify that the effects of the ZINC000014946303 and ZINC000006003042 were due to their inhibition of IGF-1R in Ewing sarcoma cells, we assessed levels of IGF-1R using Western blotting. Western blotting demonstrated that IGF-1R expression decreases with increasing drug concentrations (Figure 6F). These findings suggest that ZINC000014946303 and ZINC000006003042 induce apoptosis in Ewing sarcoma cells by inhibiting IGF-1R.

## DISCUSSION

Ewing sarcoma ranks second in the incidence of primary malignant bone tumors among children and adolescents, and its proportion in primary bone tumors is 6% to 8% [1]. After traditional surgery, radiotherapy and chemotherapy, most patients will die within 2 years, and the five-year survival rate is under 10% [2, 3]. After IGF-1R is activated and overexpressed in Ewing sarcoma, it may have a synergistic effect on the fusion gene EWS-FLI1, thus driving the occurrence of Ewing sarcoma [4, 6]. For this reason, finding the optimal IGF-1R inhibitors can provide new ideas and options for the treatment of Ewing sarcoma.

At the same time, IGF-1R was also active in some diseases. For example, IGF-1R exerts an enormous function in developing bone tumors, breast carcinoma, and colorectal cancer [8]. Several common IGF blocking methods, such as antisense RNA probe, monoclonal antibody, dominant IGF-1R negative gene mutation, and tyrosine kinase inhibitor, has good therapeutic potential in disease treatment. With the discovery of the important cancer-promoting protein IGF-1R, many targeted drugs of IGF-1R have been continuously developed and gradually applied to disease treatment [9].

IGF-1R inhibitors still have some side effects, so we need to conduct more experiments to alleviate them. For example, anti-IGF-1R monoclonal antibody therapy can only be effective in a few patients with specific tumor types such as Ewing sarcoma and thymoma. Nevertheless, in other cancer patients, anti-IGF-1R monoclonal antibodies have no significant therapeutic effect [9]. TAE226 can substantially inhibit the local growth and metastasis of primary tumors in Ewing sarcoma, which is predicted to be a candidate monotherapy or combination therapy for patients with recurrent and metastatic tumors in the future. However, TAE226 has potent cytotoxicity to Ewing sarcoma cells, which requires further study before it can be applied to the clinical treatment of Ewing sarcoma [10]. Therefore, there is an urgent need to select more targeted IGF-1R medicines for clinical application.

In this research, the virtual screening and analysis will use technical modules such as LibDock, CDOCKER, TOPKAT, etc. We choose ligands from a subset of natural products in the ZINC database's biogenic classification, and these ligands must have been named and available for purchase. We performed a virtual screening of 17931 biologically named ligands in the ZINC15 database. The selected ligands were molecularly docked with IGF-1R using the LibDock module, and the higher the LibDock score, the better the docking effect was proven. It could be seen from the results that the compounds with high LibDock fraction had more stable conformation than other compounds. In terms of the LibDock score, the compounds with the top 20 scores proceeded to the following research.

The pharmacological characteristics of candidate compounds are evaluated by ADME and toxicity indicators. ADME provides six computational models for predicting absorption, distribution, metabolism, excretion, and toxicological properties from chemical structure, enabling the prediction of potentially problematic new chemical entities at an early stage of drug discovery. ZINC000014946303 and ZINC000006003042 have good solubility in water. It is beneficial to their absorption and metabolism in the human body and exerts better medicinal effects, so they are selected for follow-up research. In addition, they have no hepatotoxicity and avoid disrupting the normal functioning of the liver. Compared with other compounds, they have less rodent carcinogenicity, Ames mutagenicity, potential characteristics of developmental toxicity, and higher safety. The reference drug ZINC38433168 also has no hepatotoxicity, with less rodent carcinogenicity and Ames mutagenicity. It is also predicted to be a non-CYP2D6 inhibitor with weak absorbability in plasma protein binding properties. Therefore, ZINC000014946303 and ZINC000006003042 are considered to be safe drug candidates. On the other hand, although the remaining drugs have certain toxicity or side effects, they may still promote drug development. Considering all results, ZINC000014946303 and ZINC000006003042 are suitable to be ideal compounds and further investigation.

The candidate compound's bonding mechanism and chemical bonds are usually investigated using the CDOCKER module. The CDOCKER potential energy of ZINC000014946303 and ZINC000006003042 was lower than ZINC38433168, as calculated by the CDOCKER module. It can be deduced that their binding affinity for IGF-1R was also higher than that of the reference drug ZINC38433168 [15].

The stability of the ligand-IGF-1R complex in the natural environment was evaluated using molecular dynamics simulations. The potential energy and RMSD images for each complex show that the tracks of ZINC000014946303 and ZINC000006003042 come into equilibrium after 20 picoseconds. In time, the potential energy and RMSD of these 2 complexes tend to be stable. It can be inferred that ZINC000014946303 and ZINC000006003042 can bind to IGF-1R to form complexes, which can exist stably in the natural environment inhibiting IGF-1R. We can proceed with further drug development and purification in light of research results.

Screening of lead compounds exerts an enormous function on drug design. We use computer technology to assist the virtual operation, which can effectively identify possible IGF-1R inhibitors. The LibDock technology is then continued and monitored for metabolism, distribution, absorption, excretion, toxicity, etc.

We performed a point mutation on the simulated structure by Schrödinger software, replacing the LYS at 1033 sites with ARG, and the results showed that the drugs could not dock with the receptor after the point mutation. We use molecular docking and molecular dynamics simulation to evaluate the binding affinity and stability of the ligand-IGF-1R. ZINC000014946303 and ZINC000006003042 presumably have a suppressive impact on Ewing sarcoma. As everyone knows, a drug needs to be refined and improved thousands of times before reaching the market standard. Hereby, we need to carry out more improvements and refinements of drugs.

Finally, this research aimed to create a database of natural chemicals to find more potential therapeutic candidates that can inhibit IGF-1R. Even though this study was meticulously planned and executed with exact measurements, we must acknowledge that it had significant shortcomings. No medicine can be sold unless it has been improved and developed. To make these two compounds more perfect as therapeutic candidates, several groups and atoms that can influence the pharmacological features of the pharmaceuticals must be adjusted. More pharmaceutical safety indicators, such as MTD (Maximum Tolerated Dosage) and AB (Aerobic Biodegradability), should be researched in the future to validate our findings. These restrictions will be the subject of our future research.

## CONCLUSIONS

This research employs a collection of computer-aided chemical and structural technologies (such as toxicity indicators, ADME, virtual screening, molecular docking, and kinetic simulations) to select and appraise lead compounds from many natural medicines that may inhibit IGF-1R function. After carefully designed and accurate calculations, both compounds ZINC000014946303 and ZINC000006003042 can be considered safe and reliable candidate drugs with the ability to competitively inhibit IGF-1R, and high affinity for IGF-1R, and slight potential for developmental toxicity. In addition, we provide a certain amount of candidate drugs with pharmacological characteristics, which promote the continuous development of the design and improvement of IGF-1R and other drugs.

## MATERIALS AND METHODS

### ZINC database and discovery studio software

Discovery Studio software is characterized by simulating small and macromolecular systems. Its primary function is to proceed with the screening, design, and modification of potential drugs by utilizing calculations of structural biology and chemistry, right after identifying and refining lead compounds and candidate drug pathways [16].

LibDock, CDOCKER, ADME, TOPKAT belong to the Discovery Studio4.5 system. The functions of each module are as follows, LibDock is a fast molecular docking tool for fast and accurate virtual screening of large-scale databases. CDOCKER is a CHARMm-based flexible docking program that uses soft-core potentials and optimization grid representation to interface ligand molecules with active receptor sites. ADME provides six computational models for predicting absorption, distribution, metabolism, excretion, and toxicological properties from chemical structure. TOPKAT is a tool for predicting the toxicological properties of compounds. The Ames test (Salmonella typhimurium reverse mutation assay) is a bacterial short-term test for identification of carcinogens using mutagenicity in bacteria as an end point. Previous researchers have conducted drug selection studies through this method to verify the reliability of these modules [17–19]. At UCSF (University of California, San Francisco), Irwin and shochet laboratories provide the ZINC15 database available for unrestricted commercial use.

### LibDock for virtual filtering

The high-throughput screening (HTS) initiation occurs in the search for IGF-1R inhibitors. HTS focuses on compounds that are in affinity to IGF-1R. Since the reference drug ZINC38433168 is produced in a complete drug library, synthetic aperture radar is established for hundreds of analogs [15]. LibDock is able to act upon the virtual filtering step [20]. It can utilize probes and grid sites to calculate hot spots in protein. Then protein hot spots are further used for ligand arrangement and intelligent minimization algorithm, and CHARMm field of force is applied to minimization of ligand so that favorable interactions can be formed [21]. All ligand positions are sorted according to the ligand fraction when the ligand is minimized. The protein database identifier of IGF-1R is 3O23, and its chemical structure is listed in Figure 2. The protein database identifier of the reference drug is ZINC38433168. We got 3O23's crystal structure from the Protein Database (PDB), and the ligand-binding region of 3O23 was chosen. It was prepared by removing the crystal water and other heteroatoms from the surrounding area, followed by protonation and hydrogen addition. Following that, the produced protein was utilized to determine the binding site, which was then picked as the best docking site. The next step is to bind the ready ligand pair to the active site and perform virtual screening. Docking sites are sorted and grouped according to the LibDock score by the name of the corresponding compound.

### Toxicity indicators and ADME characteristics of ligands

With respect to the detections of distribution and metabolic profiles, excretion and absorption levels for candidate compounds, and potential compounds' toxicity and other properties, our researchers take advantage of the ADME module and TOPKAT module in DS4.5. Apropos of BBB and PPB levels, water-solubility, CYP2D6, hepatotoxicity, Ames mutagenicity, rodent carcinogenicity, and DTP, it is essential to thoroughly investigate the aforesaid pharmacological characteristics before we can begin to select IGF-1R inhibitors.

### Molecule docking and pharmacophore prediction

When conducting molecular docking research, we apply the CDOCKER module in DS4.5. CDOCKER is characterized by the ability to analyze the posture of the molecule under test and the high precision docking with the help of the force field of CHARMm. The interaction energy generated by the force field of CHARMm acts on the receptor and ligand and is reflected in their binding affinity. When the ligand is docked with the receptor, the water molecule changes from a crystalline state to an immobilized state, continuously changing the formation state of the complex [22, 23]. It is essential to remove the water molecules and add hydrogen atoms to the protein. When the binding of ligand and receptor reaches the optimal docking state, the complex will dock with IGF-1R and then initiate the CDOCKER process [24, 25].

### Dynamic simulation experiment

For a start, we chose the optimal binding conformation of each compound to conduct molecular dynamics simulation experiments. The first step is to establish an orthogonal box that can accommodate the ligand-receptor complex and solvate it using a water model. The system was then injected with solid chloride to simulate the physiological environment, and the force field of CHARMm and energy was adjusted to a minimum. The temperature and pressure were adjusted throughout the operation of the production module, with a duration and step size of 25 ps and 1 fs. The linear constraint solving algorithm and the particle grid Ewald algorithm respectively correspond to fixation of the hydrogen bond and calculation of the long-distance electrostatic field. Ultimately, we define the initial complex settings as the reference. The trajectory's structure, energy, and RMSD can be analyzed using the DS4.5 trajectory protocol.

### Cell lines

The American Type Culture Collection provided Ewing sarcoma cells (RD-ES). The cells were cultured in Dulbecco's modified Eagle medium (DMEM, Hyclone, USA) supplemented with 10% fetal bovine serum (Gibco, MD, USA). At 37° C, the culture dishes were kept in 5% CO2 and 95% air.

### MTT assay

Ewing sarcoma cells (RD-ES) were plated at a density of 500 cells per well in 96-well culture plates and treated with different doses of ZINC000014946303 and ZINC000006003042. MTT (Sigma, St. Louis, MO, USA) was dissolved in phosphate-buffered saline (5 mg/mL) to test cell viability. The media was replaced with fresh DMEM supplemented with 10% fetal bovine serum and diluted MTT (1:10, 10% MTT) on the day of measurement, and the cells were incubated for 3.5 hours at 37° C. The formazan crystals were then dissolved in 200 L of dimethyl sulfoxide solution after removing the incubation medium. The decrease of MTT was measured using an ELx800 absorbance microplate reader (BioTek Instruments, VT, USA) based on light absorbance at 570 nm.

### Colony formation assay

At a density of 50 cells/cm^2^, Ewing sarcoma cells (RD-ES) were seeded in Petri dishes. After 24h of culture, treated the cells with different doses of ZINC000014946303 and ZINC000006003042, respectively. Colonies were counted and characterized after 10 days of *in vitro* culture. Then rinsed the colonies with phosphate-buffered saline, fixed the 4% paraformaldehyde for 15 min, added 5% crystal violet staining for 30 min after discarding the fixative solution, and slowly washed off the staining solution with running water.

### *In vitro* scratch assay

RD-ES cells were first cultured on 24-well Permanox™ plates until the entire plate was covered. Used a 10-μL pipette tip to evenly stroke consistent cell-free areas in each well along a ruler and slowly rinse loose cells using DMEM. Cells were then treated with different doses of ZINC000014946303 and ZINC000006003042. An image of the scraped area was captured using a phase-contrast microscope at three-time points of 0, 12, and 24 hours. The remaining wounded areas and scratch width were measured at six different points in each image.

### Western blotting

Ewing sarcoma cells (RD-ES) in the log growth phase were seeded at a density of 2 × 10^5^ cells/well in a six-well plate, followed by treatment with different doses of ZINC000014946303 and ZINC000006003042. After 48 h of culture, the cells were lysed to extract the total protein and electrophoretically separated. Transferred the protein onto the membranes, cut the protein band of interest, treated it with primary antibodies against IGF-1R and β-actin, then washed the membranes and incubated with secondary antibodies. The membranes were visualized by an enhanced chemiluminescence detection system (Pierce; Thermo Fisher Scientific, Inc.).
